# A Rare Case of Atypical Hemolytic Uremic Syndrome Presenting as Chronic Interstitial Nephritis

**DOI:** 10.7759/cureus.65274

**Published:** 2024-07-24

**Authors:** Shruthi Muralidharan, Gerry G Mathew, Anand Alwan, Varadharajan Jayaprakash

**Affiliations:** 1 Nephrology, SRM Medical College Hospital and Research Centre, Chengalpattu, IND; 2 Radiology, SRM Global Hospitals, Chennai, IND

**Keywords:** cfhr3 gene, complement dysregulation, posterior reversible encephalopathy syndrome, chronic interstitial nephritis, atypical haemolytic uremic syndrome

## Abstract

Atypical hemolytic uremic syndrome commonly presents as rapidly progressive renal failure and is histologically characterized by thrombotic microangiopathy (TMA). TMA presenting with acute renal failure requires aggressive medical management. Here, we present a case of a 30-year-old man who presented with a history of accelerated hypertension and a strong family history of end-stage renal disease, in September 2023. Upon evaluation, he was found to have a creatinine level of 2 mg/dl, bland urine and normal-sized kidneys; a renal biopsy revealed chronic interstitial nephritis. Genetic analysis for autosomal dominant tubulointerstitial kidney disease and nephronophthisis yielded negative results. The patient was managed with antihypertensive medications. In January 2024, he was admitted with a history of confusion, headache, and alcohol binge. He had a blood pressure of 200/100 mmHg and had grade 3 hypertensive retinopathy. Laboratory tests revealed anemia with thrombocytopenia, bland urine, normal coagulation parameters, indirect hyperbilirubinemia, normal-sized kidneys on ultrasound, and elevated lactate dehydrogenase levels. MRI of the brain revealed symmetrical hyperintensities in bilateral cerebellum and the dorsal brainstem. Complement levels revealed low C3 levels and genetic analysis revealed a homozygous deletion in the complement factor H-related 3 (CFHR3) gene. The autoantibody for complement factor H was negative. The patient was managed conservatively with adequate blood pressure control. This case highlights the effects of complement dysregulation on the renal tubulointerstitium.

## Introduction

Atypical hemolytic uremic syndrome (aHUS) is a rare complement-mediated renal disorder characterized by thrombotic microangiopathy (TMA) of the glomerular vessels and thrombosis of the renal arterioles [1]. aHUS is typically characterized by thrombocytopenia, hemolytic anemia, and acute kidney injury, with an annual incidence of one to two cases per million population [1]. aHUS majorly occurs in childhood and adolescence, with a 60%-80% probability of progression to end-stage renal disease (ESRD) [1,2]. In contrast, typical HUS is caused predominantly by Shiga toxin-producing *Escherichia coli* (STEC) with overlapping clinical features, and has a possibility of 85% recovery of kidney function [1,2]. TMA associated with aHUS needs to be tackled with aggressive plasma exchange and eculizumab. Renal lesions commonly described with aHUS include TMA and renal-limited C3 glomerulopathy [1,2]. There are sparse medical case reports on the intricate effects of complement dysregulation on renal interstitium, its clinical presentation and management strategies. This case report describes a rare renal interstitial lesion associated with aHUS, with atypical neuro-radiological findings.

## Case presentation

**Figure 1:**
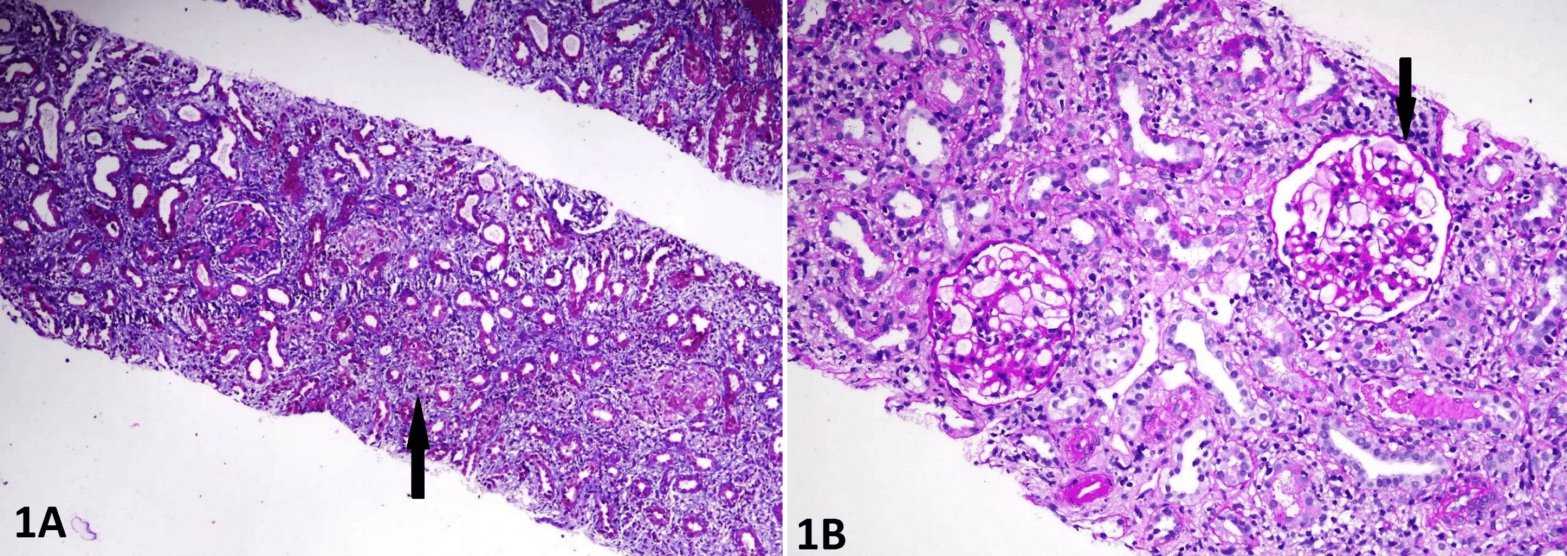
(A) Masson’s trichrome stain of the renal biopsy specimen showing advanced interstitial fibrosis and tubular atrophy (black arrow) having lymphocytic infiltrate interspersed with sclerotic and viable glomeruli (magnification: 40x; 600 DPI). (B) Periodic acid-Schiff stain of the renal biopsy specimen showing two viable glomeruli with normal cellularity and patent capillary lumens (black arrow) with chronic lymphocytic infiltrate in the interstitium (magnification: 100x; 600 DPI)

A 30-year-old man presented to our hospital with a history of accelerated hypertension in September 2023. His family history included ESRD in the mother at 37 years of age and grandmother at the age of 41 years. Clinically, the patient had mild pallor and tachycardia without any significant systemic abnormalities. Laboratory investigations revealed a creatinine level at 2 mg/dl, hemoglobin at 9 g/dl, platelets at 129,000/mm^3^, bland urine and normal-sized kidneys on ultrasound. A renal biopsy was performed in view of persistent azotemia, which revealed six glomeruli with three sclerotic glomeruli. Viable glomeruli were normocellular with patent capillary lumens, and lymphocytic infiltrate was interspersed in the fibrotic interstitium with 50% interstitial fibrosis and tubular atrophy (IFTA), suggesting chronic interstitial nephritis (Figures 1A-1B).

**Figure 2:**
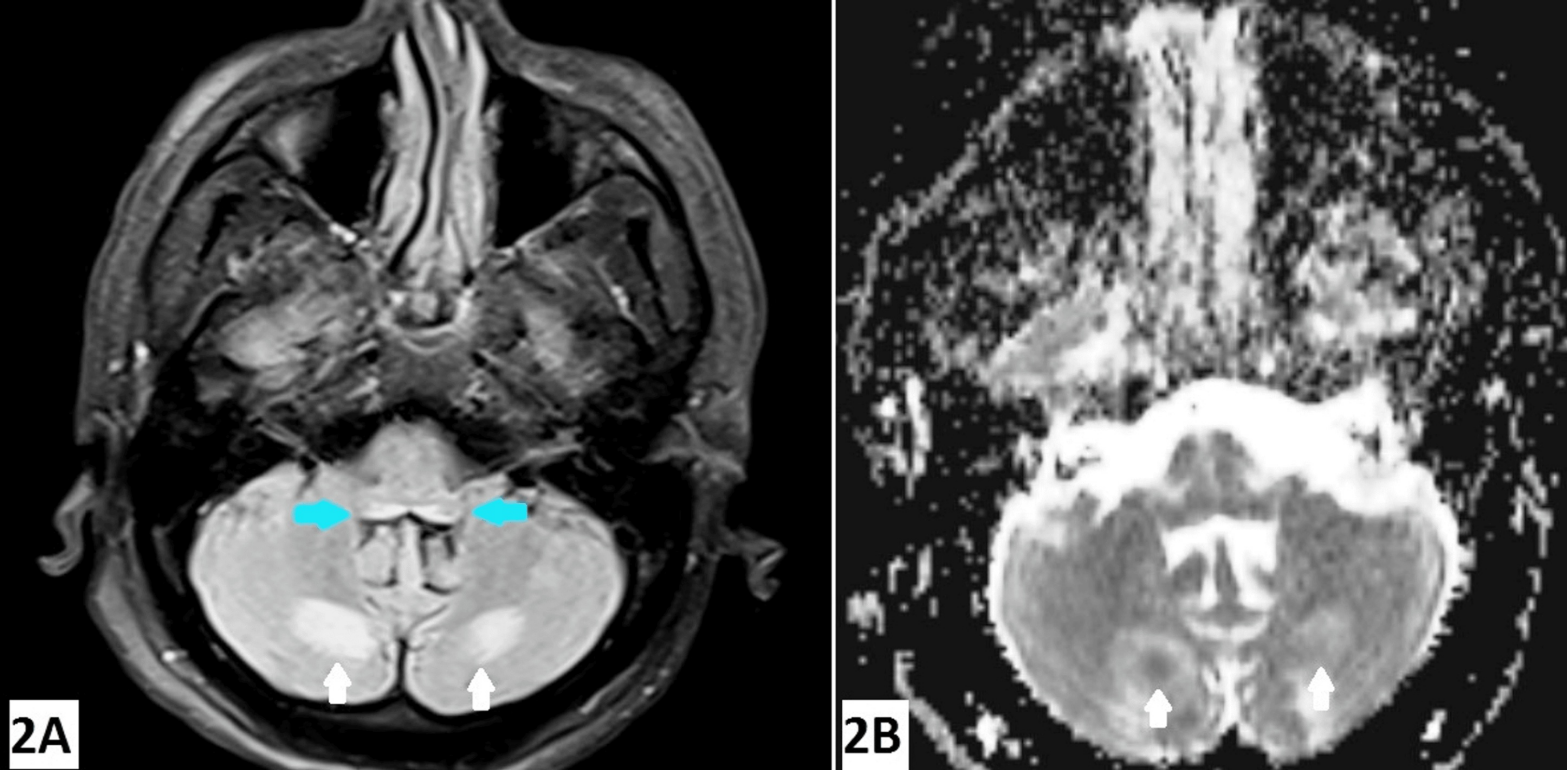
(A) Brain MRI (T2 FLAIR) showing areas of symmetrical FLAIR hyperintensities in an area posterior to the cerebellar dentate nuclei (white arrows) and dorsal aspect of the upper medulla (blue arrows) (magnification: 600 DPI). (B) Brain MRI ADC sequence showing areas of low ADC bilaterally in an area posterior to the cerebellar dentate nuclei (white arrows) (magnification: 600 DPI) FLAIR, fluid-attenuated inversion recovery; ADC, apparent diffusion coefficient

Focused genetic analysis for nephronophthisis and autosomal dominant tubulointerstitial kidney disease yielded negative results. The patient was managed with antihypertensive medications and regularly followed up. He presented with a history of new-onset confusion, and headache without ataxia or ophthalmoplegia in January 2024. A significant history included an alcoholic binge two days prior to the index event. A clinical examination revealed mild confusion without any focal neurological deficits and grade 3 hypertensive retinopathy with a blood pressure of 200/100 mm Hg. Wernicke’s encephalopathy was clinically suspected based on an alcohol binge, and the patient was managed with thiamine injections. Brain MRI revealed areas of fluid-attenuated inversion recovery (FLAIR) hyperintensity (Figure 2A) with central foci of diffusion restriction and corresponding low signal on the apparent diffusion coefficient (ADC) sequence (Figure 2B) just posterior to the cerebellar dentate nuclei and FLAIR hyperintensity with no corresponding diffusion restriction in the dorsal aspect of the upper medulla.

Labs revealed anemia, thrombocytopenia, elevated lactate dehydrogenase (LDH) levels, indirect hyperbilirubinemia and normal coagulation parameters (Table 1).

**Table 1:** Laboratory parameters (January 2024)

Parameters	Values	Reference range
Hemoglobin(g/dl)	8.2	13-16
White blood cell count (/mm^3^)	6840	4000-11,000
Platelets (/mm^3^)	131,400	150,000-410,000
Serum creatinine (mg/dl)	2.7	0.6-1.2
Serum urea (mg/dl)	41	15-45
Serum total bilirubin (mg/dl)	2	0.3-1
Indirect bilirubin (mg/dl)	1.6	0.2-0.8
Aspartate transaminase (IU/l)	52	8-45
Alanine aminotransferase (IU/l)	34	10-33
Prothrombin time (test-sec)	13	<12
INR	1.08	<1.1
C3 (mg/dl)	78	90-180
C4 (mg/dl)	28	10-40
Lactate dehydrogenase (IU/l)	351	140-280

Abdominal computed tomography revealed normal-sized kidneys. A peripheral smear revealed normocytic anemia, tear drop cells without schistocytes; the Coombs test was negative. Vitamin B-12 and folate levels were normal. The urine examination revealed bland urine without proteinuria or hematuria. In view of thrombocytopenia, accelerated hypertension, raised LDH levels, and background azotemia, complement levels were measured, which revealed depressed C3 levels and normal C4 levels (Table 1). In the background of depressed C3, thrombocytopenia, a history of accelerated hypertension, and early-onset chronic kidney disease in three generations, whole exome next-generation sequencing with in silico analysis was done that revealed homozygous deletion in the complement factor H-related 3 (CFHR3) gene [1(g.(196774945_196779161)_(196794074_196793316)del)]. The autoantibody for factor H was negative. A renal biopsy was not reattempted due to patient's non-consent and because the previous biopsy had already revealed chronic interstitial nephritis with advanced interstitial fibrosis and tubular atrophy. The patient was managed conservatively; confusion resolved completely, thrombocytopenia resolved gradually by day 6 post-admission, and the creatinine level stabilized to the baseline level of 2 mg/dl on discharge.

## Discussion

aHUS is a low-penetrant, complement-mediated disorder characterized by thrombocytopenia, microangiopathic hemolytic anemia, and acute kidney injury with an incidence of one to two cases per million [1]. Renal histology described in aHUS in the medical literature is classically thrombotic microangiopathy within glomerular capillaries, with a few exceptions of renal-limited C3 glomerulopathy [1]. Genetic defects in aHUS include loss-of-function mutations in complement regulator genes (CFH, CD46, thrombomodulin (THBD), and complement factor I) or gain-of-function mutations in genes such as C3 and complement factor B [1,2]. The medical literature classically describes an established association between homozygous gene deletion in CFHR1-3 and antibodies to complement factor H (anti-CFH) in aHUS. Our case is unique in that there was a homozygous deletion in the CFHR3 gene that presented as biopsy-proven chronic interstitial nephritis with a strong family history of ESRD.

Complement activation causing glomerular lesions such as thrombotic microangiopathy is well established; however, the literature surrounding the pathogenesis of complement-mediated tubulointerstitial damage is limited [3]. In aHUS, due to unregulated complement activation caused by genetic defects, it is observed that plasma C3 binds to the amine or carbohydrate groups present on the basolateral aspect of the tubules via its thioester linkage, leading to the activation of complement fragments [4]. This dysregulated activation of complement fragments induces intrinsic and extrinsic inflammation in the form of macrophages and dendritic cells, culminating in direct cellular injury and interstitial damage [4]. This experimental evidence of tubulointerstitial damage induced by an overactive complement system has been elegantly described in mouse models lacking the Crry protein, a transmembrane molecule analogous to the membrane cofactor protein (MCP) in humans, whose defects contribute to the pathogenesis of aHUS [4].

The neurological manifestations of aHUS include encephalopathy, seizures, confusion, stupor, and coma [5]. The classical findings of aHUS on brain MRI include symmetrical hyperintensities in the basal ganglia with involvement of the dorsolateral aspect of the lentiform nucleus [6]. However, in our case, hyperintensities were observed bilaterally in the cerebellum and dorsal brainstem. At the same time, the possibility of aHUS cannot be completely ruled out because there are descriptions of hyperintensities in the thalami, cerebellum, centrum semiovale, brainstem, and commissural fibres in the literature associated with aHUS [5,6]. Considering the background of accelerated hypertension, grade 3 hypertensive retinopathy, and confusion with new-onset headache, the possibility of posterior reversible encephalopathy syndrome (PRES) was also considered. The characteristic radiological findings of PRES include hyperintensities predominantly in the parieto-occipital region; however, the literature mentions isolated cases of hyperintensities in the cerebellum, brainstem, and deep white matter [6]. This clinical diagnosis is plausible because the patient’s clinical condition improved with adequate blood pressure control without the need of complement inhibitors like eculizumab. Wernicke’s encephalopathy was unlikely in our case because, radiologically, brain MRI did not demonstrate symmetrical hyperintensities in the mammillary bodies, thalami, periaqueductal area, or tectal plate [7]. The differential diagnosis of hyperintensities in the cerebellum and brainstem includes brain stem injury, Behcet’s disease, hypertensive brain stem encephalopathy, PRES, aHUS, and multisystem atrophy [5,6,8].

A plausible explanation for the absence of anti-CFH in our patient is that the mutation affected the CFHR3 gene, and anti-CFH was frequently encountered with gene mutations encoding the CFHR1 protein [9]. The mere presence of anti-CFH is not sufficient for the florid presentation of aHUS, but multiple hits are required for full-blown thrombotic microangiopathy [9], which could explain the indolent nature of the disease in our patient. We postulated the possibility of repeated submaximal antigenic stimulation for complement activation in the fluid phase [4,10], which would have triggered macrophages and dendritic cells, thereby leading to cumulative interstitial injury manifesting as chronic interstitial nephritis with advanced IFTA. The level of complement activation triggered by multiple antigenic hits determines the clinical severity of aHUS [9,10]; hence, we believe that the utility of eculizumab in subtle atypical HUS will be determined by complement activation patterns and the level of activation as evidenced by alternate complement pathway activation markers [10]. The absence of hematuria and near-stable azotemia clearly indicates the absence of vascular involvement.

A limitation of this case report is the lack of renal re-biopsy that could have shed more light on this atypical renal presentation. This case report doesn’t stress on the pathogenic nature of the mutation or the atypical neurological presentation but emphasizes the variable clinical presentation in complement dysregulation.

The differential diagnosis of aHUS includes typical hemolytic uremic syndrome caused by STEC (diarrhea-associated HUS) and thrombotic thrombocytopenic purpura (TTP) [11]. STEC-associated HUS is characterized by abdominal pain, bloody diarrhea, and renal failure due to the Shiga toxin, which is managed by volume expansion and renal replacement as clinically indicated [12]. There is controversy regarding the use of antibiotics in STEC-associated HUS, predominantly due to the release of the Shiga toxin from dead bacteria, thereby aggravating the clinical condition [11]. TTP is characterized by von Willebrand factor (VWF) platelet thrombi in the arterioles and capillaries of multiple organs due to the deficiency of a disintegrin and metalloproteinase with a thrombospondin type 1 motif, member 13 (ADAMTS13) [11]. TTP and aHUS are clinically distinguished by predominant neurological manifestations in TTP and predominant renal manifestations in aHUS, with other overlapping clinical features. TTP diagnosis involves documenting ADAMTS13 deficiency using specific assays, and treatment involves plasmapheresis [12]. The treatment of aHUS involves plasmapheresis with the addition of eculizumab, a monoclonal antibody that targets complement protein C5 [1,2,12]. Since post-transplant recurrence is high, most cases of aHUS with renal involvement are managed by combined liver kidney transplantation, except in cases where MCP mutations exist [1,2,12]. The advent of eculizumab has revolutionized the possibility of isolated renal transplantation with better graft outcomes in the early initiation of eculizumab treatment arm compared to the late initiation of eculizumab post-renal transplant [12].

This case explores the pathological impact of unregulated complement activation on the tubulointerstitial compartment; it highlights the importance of considering this rare diagnosis in the clinical background of a strong family history of early-onset ESRD and opens new research avenues to investigate the effect of complement activation on the interstitial compartment leading to indolent chronic kidney disease.

## Conclusions

Complement dysregulation involving the CFHR3 gene mutation responsible for aHUS traditionally encompasses the histopathological picture of thrombotic microangiopathy and C3 glomerulopathy, and usually presents with rapidly progressive renal failure. The effect of CFHR3 gene mutation on renal interstitial compartment presenting as chronic interstitial compartment should be considered in cases of young-onset chronic kidney disease with a strong family history of ESRD and low C3 levels.

## References

[1] Zhang K, Lu Y, Harley KT, Tran MH (2017). Atypical hemolytic uremic syndrome: a brief review. Hematol Rep.

[2] Józsi M, Licht C, Strobel S (2008). Factor H autoantibodies in atypical hemolytic uremic syndrome correlate with CFHR1/CFHR3 deficiency. Blood.

[3] Fearn A, Sheerin NS (2015). Complement activation in progressive renal disease. World J Nephrol.

[4] Brar JE, Quigg RJ (2014). Complement activation in the tubulointerstitium: AKI, CKD, and in between. Kidney Int.

[5] Yerigeri K, Kadatane S, Mongan K (2023). Atypical hemolytic-uremic syndrome: genetic basis, clinical manifestations, and a multidisciplinary approach to management. J Multidiscip Healthc.

[6] Mansour MA, Khalil DF, Hasham MA, Youssef A, Rashad M, Awadallah M, Ali H (2023). Hemolytic uremic syndrome with central nervous system manifestations, a case report and literature review. Radiol Case Rep.

[7] Zuccoli G, Santa Cruz D, Bertolini M, Rovira A, Gallucci M, Carollo C, Pipitone N (2009). MR imaging findings in 56 patients with Wernicke encephalopathy: nonalcoholics may differ from alcoholics. AJNR Am J Neuroradiol.

[8] Guzmán-De-Villoria JA, Ferreiro-Argüelles C, Fernández-García P (2010). Differential diagnosis of T2 hyperintense brainstem lesions: Part 2. Diffuse lesions. Semin Ultrasound CT MR.

[9] Moore I, Strain L, Pappworth I (2010). Association of factor H autoantibodies with deletions of CFHR1, CFHR3, CFHR4, and with mutations in CFH, CFI, CD46, and C3 in patients with atypical hemolytic uremic syndrome. Blood.

[10] Volokhina EB, Westra D, van der Velden TJ, van de Kar NC, Mollnes TE, van den Heuvel LP (2015). Complement activation patterns in atypical haemolytic uraemic syndrome during acute phase and in remission. Clin Exp Immunol.

[11] Yenerel MN (2014). Atypical hemolytic uremic syndrome: differential diagnosis from TTP/HUS and management. Turk J Haematol.

[12] Siedlecki AM, Isbel N, Vande Walle J, James Eggleston J, Cohen DJ (2019). Eculizumab use for kidney transplantation in patients with a diagnosis of atypical hemolytic uremic syndrome. Kidney Int Rep.

